# Which method is optimal for estimating variance components and their variability in generalizability theory? evidence form a set of unified rules for bootstrap method

**DOI:** 10.1371/journal.pone.0288069

**Published:** 2023-07-14

**Authors:** Guangming Li

**Affiliations:** School of Psychology, Center for Studies of Psychological Application, Guangdong Key Laboratory of Mental Health and Cognitive Science, South China Normal University, Guangzhou, 510631, China; Zhejiang Normal University, CHINA

## Abstract

**Objective:**

The purpose of this study is to compare the performance of the four estimation methods (traditional method, jackknife method, bootstrap method, and MCMC method), find the optimal one, and make a set of unified rules for Bootstrap.

**Methods:**

Based on four types of simulated data (normal, dichotomous, polytomous, and skewed data), this study estimates and compares the estimated variance components and their variability of the four estimation methods when using a *p×i* design in generalizability theory. The estimated variance components are vc.p, vc.i and vc.pi and the variability of estimated variance components are their estimated standard errors (SE(vc.p), SE(vc.i) and SE(vc.pi)) and confidence intervals (CI(vc.p), CI(vc.i) and CI(vc.pi)).

**Results:**

For the normal data, all the four methods can accurately estimate the variance components and their variability. For the dichotomous data, the |RPB| of SE (vc.i) of traditional method is 128.5714, the |RPB| of SE (vc.i), SE (vc.pi) and CI (vc.i) of jackknife method are 42.8571, 43.6893 and 40.5000, which are larger than 25 and not accurate. For the polytomous data, the |RPB| of SE (vc.i) and CI (vc.i) of MCMC method are 59.6612 and 45.2500, which are larger than 25 and not accurate. For the skewed data, the |RPB| of SE (vc.p), SE (vc.i) and SE (vc. pi) of traditional method and MCMC method are over 25, which are not accurate. Only the bootstrap method can estimate variance components and their variability accurately across different data distribution. Nonetheless, the divide-and-conquer strategy must be used when adopting the bootstrap method.

**Conclusions:**

The bootstrap method is optimal among the four methods and shows the cross-distribution superiority over the other three methods. However, a set of unified rules for the divide-and-conquer strategy need to be recommended for the bootstrap method, which is optimal when boot-p for *p* (person), boot-pi for *i* (item), and boot-i for *pi* (person × item).

## 1 Introduction

Generalizability theory (G theory) is widely used in psychological and educational measurement [1–3]. In G theory, estimating variance components (VC) is the essential technique and is of central importance. However, these estimates are, like any statistic, subject to sampling variability. They are likely to vary from one sample to another. Therefore, estimating the variability of estimated variance components needs to be further explored in order to ensure the dependability of estimated variance components. Brennan (2001) [4] thought that we should pay attention to the influence of sampling errors on variance components’ estimation, and it is the “Achilles heel” to estimate the variability of estimated variance components.

Compared with classical test theory, G theory has an advantage of providing a conceptual and statistical approach to estimate variance components with relatively high precision [5]. By identifying, decomposing, and examining sources of measurement errors, G theory makes it possible to reduce measurement error variances [6]. As a variance components model, G theory has a focus on variance components estimation [4,7]. The accuracy and reliability of variance components estimates in G theory can be explained by the Generalizability study (G study) [8]. In order to minimize estimation error, multiple independent repeated measurements would be an ideal option [4], yet this method is somehow impractical for the measurement procedure which is administered only once in many cases. Furthermore, the estimated variance components may also vary depending on the sample size. For instance, a small sample will lead to large estimation errors, especially in a complicated design, such as the multifaceted G study mixed design [9–11].

The estimation of the variability of estimated variance components using G theory can be addressed by calculating the standard errors (SE) and confidence intervals (CI) of the estimates. However, the standard errors and confidence intervals of estimated variance components are affected by factors such as estimation methods [12,13], types of data (e.g., distributions) [14], and model effects [15,16]. G theory allows both approximate estimation methods (e.g., traditional method and MCMC method) and resampling methods (e.g., jackknife method and bootstrap method). Nevertheless, few published studies have compared the performance of different estimation methods for estimating standard errors and confidence intervals of variance components. Little is known regarding whether or not one method is superior to the others. If there is an optimal method, then a set of unified rules will be of great utility. Besides, non-normal distributions data have yet been less taken into account when estimating the variability [17–19].

Some researchers have developed and recommended unified rules for estimating the standard errors of variance components [13]. Taking the *p×i* design (*p* designates person or examinee and *i* indexes item) as an example, it is recommended that the some bootstrap strategies should be used for estimating the standard errors of estimated variance components (e.g., the boot-p strategy should be used for estimating the standard errors of estimated variance components of *p*). For other generalizability designs, similar unified rules should been recommended. However, these recommended unified rules have not been fully tested and were not used in different data types. Furthermore, previous literature has not indicated any unified rule for the confidence intervals of variance components’ estimates [20,21]. Indeed, it is highly challenging to provide a set of effective unified rules for the estimation of the confidence intervals of variance components, especially in complex generalizability designs.

Generally, there are a lot of things that follow the normal distribution, such as height, weight, intelligence, etc. However, non-normal distribution data is common in psychological and educational measurement practice. For example, there are only two kinds of scores (wrong and right) for multiple-choice questions and yes no questions in some exams: 0 and 1, which are dichotomous distribution data. For example, in some psychological and educational tests, the rating has multiple data points and multiple scores exist such as a score of 0–4 [22]. The score can be divided into five points, namely 0, 1, 2, 3, and 4, which is a polytomous distribution data. For skewed distribution data, it is also common in practice because with the development of society, the application fields of psychological and educational measurement have undergone significant changes, and the knowledge and abilities of the tested group are no longer subject to normal distribution and are subject to skewed distribution to a certain extent [23]. These four types of data (i.e., normal, dichotomous, polytomous, and skewed data) are more commonly used in the practical application.

The present study aims to address this problem. It is designed to compare the performance of four estimation methods (i.e., traditional, jackknife, bootstrap and MCMC method) in estimating the standard errors and confidence intervals of estimated variance components with four types of data (i.e., normal, dichotomous, polytomous, and skewed data) in a *p×i* design and to propose a unified formulation for these estimations. Specifically, we try to figure out whether any one of the estimation methods has an advantage over the other methods. If an optimal method does exist, then we will explore whether there is a set of unified rules for estimated standard errors and confidence intervals of these estimates.

## 2 Methods

### 2.1 Simulation design

Simulation design is 4*×*4×3.

Three independent variables: (1) the first 4 represents four estimation methods (traditional method, jackknife method, bootstrap method and MCMC method); (2) the second 4 represents four distribution data (normal data, dichotomous data, polytomous data, and skewed data); (3) the 3 represents three measurement effects (person, item, and person×item).

Three dependent variables: variance components, standard errors of variance components (SE(vc)), and confidence intervals of variance components (CI(vc)).

### 2.2 Estimation methods

#### 2.2.1 Traditional method

The traditional method to estimate standard errors of estimated variance components assumes that score effects have a multivariate normal distribution. Under this assumption, it can be shown that an estimator of the standard error of an estimated variance component is

$$\hat{\sigma }[{\hat{\sigma }}^{2}(\alpha |M)]=\sqrt{\sum_{\beta }\frac{2[f{\left(\beta |\alpha )MS(\beta )\right]}^{2}}{df(\beta )+2}}$$ (1)

Where *M* designates the model, *α* indexes an effect; *β* indexes the mean squares that enter ${\hat{\sigma }}^{2}(\alpha |M)$, and *f*(*β*|*α*) is the coefficient of *MS*(*β*) in the linear combination of mean squares that gives ${\hat{\sigma }}^{2}(\alpha |M)$. The square of the right-hand side of Formula (1) is an unbiased estimator of the variance of ${\hat{\sigma }}^{2}(\alpha |M)$ [4].

When the score effect of large sample size is subject to the assumption of multivariate normal distribution for variance components, the following formula can be given to estimate the confidence interval [4]:

$${\hat{\sigma }}^{2}(\alpha |M)\pm z\hat{\sigma }[{\hat{\sigma }}^{2}(\alpha |M)]$$ (2)

Where ${\hat{\sigma }}^{2}(\alpha |M)$ is assumed to follow the normal distribution. The z represents the z score of the standard normal distribution (such as 1.96 or 2.58). $\hat{\sigma }[{\hat{\sigma }}^{2}(\alpha |M)]$ represents the standard error of ${\hat{\sigma }}^{2}(\alpha |M)$. Formula (2) is the traditional method’s formula for estimating the confidence intervals of estimated variance components.

#### 2.2.2 Jackknife method

Quenouille (1949) [24] suggested a nonparametric estimator of bias. Tudey (1958) [25] extend Quenouille’s idea to a nonparametric estimator of the standard error of a statistic. The theory underlying the jackknife is discussed extensively by Li and Zhang (2012) [14]. Here, we briefly outline the basics of the theory and then discuss its application to estimated variance components for the design.

Suppose a set of *S* data points is used to estimate some parameter *θ*. The general steps in using the jackknife to estimate the standard error of *θ* are:

1. Obtain $\hat{\theta }$ for all *S* data points;
2. Obtain the *S* estimates of *θ* that result from deleting each one of the data points, and let each such estimate be designated ${\hat{\theta }}_{-j}$.
3. For each of data points, obtain ${\hat{\theta }}_{*j}=\hat{\theta }+(s-1)(\hat{\theta }-{\hat{\theta }}_{-j})$, which is called “pseudovaluses”.
4. Obtain the mean of the pseudovaluses ${\hat{\theta }}_{J}$, which is the jackknife estimator of *θ*;
5. Obtain the jackknife estimate of the standard error of $\hat{\theta }$:

$$\hat{\sigma }({\hat{\theta }}_{J})=\sqrt{\frac{1}{s(s-1)}\sum_{j=1}^{s}{\left({\hat{\theta }}_{*j}-{\hat{\theta }}_{J}\right)}^{2}}$$ (3)

Which is the standard error of the mean for the pseudovaluses.

To establish a confidence interval using the jackknife, typically a distributional-form assumption is required. Usually, normality is assumed, and Student’s *t* distribution is employed. Thus, a 100(1−*α*)% confidence interval for *θ* is

$${\hat{\theta }}_{J}-t\hat{\sigma }({\hat{\theta }}_{J})\leq \theta \leq {\hat{\theta }}_{J}+t\hat{\sigma }({\hat{\theta }}_{J})$$ (4)

Where *θ* can be any one of the variance components and *t* is the (1−*α*)/2 percentile point of the *t* distribution with *n*_*p*_*n*_*i*_−1 degrees of freedom.

#### 2.2.3 Bootstrap method

The bootstrap is similar to the jackknife in that both are resampling methods and both are primarily nonparametric methods for assessing the accuracy of a particular $\hat{\theta }$ as an estimate of *θ*. A principal difference between the two methods is that the bootstrap employs sampling with replacement, whereas the jackknife employs sampling without replacement. Efron (1982) [26] provides an early theoretical treatment of the bootstrap.

For a statistic based on *S* observations, the bootstrap algorithm is based on multiple bootstrap samples, with each such sample consisting of a random sample of size *S* with replacement from the original sample. Using the bootstrap, estimation of the standard error of a statistic $\hat{\theta }$ involves these steps [27]:

1. Using a random number generator, independently draw a large number of bootstrap samples, say *B* of them;
2. For each sample, evaluate the statistic of interest, say ${\hat{\theta }}_{b}$ (*b* = 1, 2, …,*B*);
3. Calculate the sample standard deviation of the ${\hat{\theta }}_{b}$:

$$\hat{\sigma }({\hat{\theta }}_{b})=\sqrt{\frac{\sum_{b=1}^{B}{\left({\hat{\theta }}_{b}-{\hat{\theta }}_{B}\right)}^{2}}{B-1}}$$ (5)

Where ${\hat{\theta }}_{B}=\sum_{b=1}^{B}{\hat{\theta }}_{b}/B$ is the bootstrap estimate of *θ*.

An appealing characteristic of the bootstrap algorithm is that it can be used almost automatically to obtain an approximate confidence interval, provided that the number of bootstrap sample is *B* ≥ 1000 [12]. For example, a simple approach to obtaining an 80% approximate confidence interval for *θ* is to use the 10^th^ and 90^th^ percentile points of the distribution of the $\theta_{b}$.

#### 2.2.4 MCMC method

The Markov chain Monte Carlo (MCMC) procedure is a method of simulating random samples from any theoretical distribution, especially from the multivariate posterior distribution to estimate features of the theoretical distribution [28]. The essential idea of MCMC is to define a Markov chain and to draw samples sequentially from the Markov chain. For Bayesian inference, the Markov chain is defined in such a way that the stationary distribution turns out to be the posterior distribution of interest. The draws form a Markov chain in that the distribution of the sampled draws depends only on the last value drawn. If the procedure works well, the approximate distributions are improved each iteration, which finally converge to the target distribution.

In generalizability theory, the linear model for a p×i design can be written as

$$X_{pi}=\mu +\pi_{p}+\beta_{i}+\varepsilon_{pi}$$ (6)

where *μ* refers to the grand mean; *π*_*p*_, *β*_*i*_, and *ε*_*pi*_ refer to person effect, item effect and person×item effect (including residual effect) respectively. An observed score can be viewed as two parts. One part is a linear combination of the grand mean, the person effect and the item effect. The other part is the residual effect, which could be assumed to follow a normal distribution. In Bayesian analysis, we can assign priors to the distributions of the person and item effects. If normal distributions are assumed for both effects, the model could be written as *μ*_*pi*_ = *μ*+*π*_*p*_+*β*_*i*_, with priors

$$X_{pi}|\mu_{pi},\sigma_{pi}^{2}∼N(\mu_{pi},\sigma_{pi}^{2})$$ (7)

$$p|\mu_{p},\sigma_{p}^{2}∼N(\mu_{p},\sigma_{p}^{2})$$ (8)

$$i|\mu_{i},\sigma_{i}^{2}∼N(\mu_{i},\sigma_{i}^{2})$$ (9)

In estimating the variability of estimated variance components, we are interested in the posterior means, posterior standard errors and credible sets for ${\hat{\sigma }}_{p}^{2}$, ${\hat{\sigma }}_{i}^{2}$, and ${\hat{\sigma }}_{pi}^{2}$. In order to obtain these, we need to specify priors for ${\hat{\sigma }}_{p}^{2}$, ${\hat{\sigma }}_{i}^{2}$, and ${\hat{\sigma }}_{pi}^{2}$, which are also called hyper priors. In this study, we refer to Mao et al. (2005) [29] practice, set *p~τ*(2,4), *i~τ*(2,16), *pi~τ*(2,64), and the initial value are 0.001.

### 2.3 Distribution data

Based on the *p×i* design in generalizability theory, Monte Carlo stimulation technique is used to generate four types of data with statistical software *R* (*R* 2.13.0), including normal data, dichotomous data, polytomous data, and skewed data. The simulation procedures are as follows.

#### 2.3.1 Normal data

The procedure of simulating normally distributed data followed three steps [4]. First of all, the following formula $X_{pi}=\mu +(\mu_{p}-\mu )+(\mu_{i}-\mu )+(X_{pi}-\mu_{p}-\mu_{i}+\mu )$ was transformed into $X_{pi}=\mu +\sigma_{p}z_{p}+\sigma_{i}z_{i}+\sigma_{pi}z_{pi}$. Secondly, within *R*, the rnorm function was called to randomly generate *z*_*p*_, *z*_*i*_ and *z*_*pi*_ that obeyed the normal distribution when the parameters *σ*_*p*_, *σ*_*i*_ and *σ*_*pi*_ were specified as 2, 4, and 8 (usually *μ* was set as 0). Last but not least, simulated data *X*_*pi*_ was obtained. The number of simulations was 1000. Thus, 1000 batches of 100×20 simulated normal data were generated.

#### 2.3.2 Dichotomous data

As soon as the simulated normal data *X*_*pi*_ had been obtained (see above), *Y*_*pi*_ was judged. If *X*_*pi*_≥ 0, then *Y*_*pi*_ = 1, otherwise *Y*_*pi*_ = 0. In this study, *Y*_*pi*_ obeyed Bernoulli distribution and the success probability of *Y*_*pi*_ was 0.5, which was $P(Y_{pi}=0)=1-P(X_{pi}\geq 0)=0.5$. The simulation process was conducted for 1000 times, generating 1000 batches of 100×20 dichotomous data.

#### 2.3.3 Polytomous data

The process of generating polytomous data was as follows. First of all, following the recommendations of Lane et al. (1996) [30], several parameters were obtained, including *BIN*(1,0.14715), *BIN*(1,0.0595) and *BIN*(5,0.5917). The success probability of Bernoulli distribution functions *BIN*(1,0.14715) and *BIN*(1,0.0595) for one test were 0.14715 and 0.0595 respectively, and the success probability of Bernoulli distribution function *BIN*(5,0.5917) for five tests was 0.5917. Sceondly, the rbinom function was called in *R* to generate *BIN*(1,0.14715), *BIN*(1,0.0595) and *BIN*(5,0.5917). Last but not least, polytomous distributed data were obtained using the formula provided by Li and Zhang (2012) [14]:

$$X_{pi}=2BIN(1,0.14715)+2BIN(1,0.0595)+BIN(5,0.5917)$$ (10)

Where *X*_*pi*_ had 10 points: 0, 1, 2, 3, 4, 5, 6, 7, 8, and 9. The number of simulations was 1000, which produced 1000 batches of 100×20 polytomous data.

#### 2.3.4 Skewed data

It took three steps to simulate skewed data. In step one, the generalized hyperbolic distribution was defined, which was referred to as GH distribution. The density function of GH distribution [31,32] was

$$GH(x;\lambda ,\alpha ,\beta ,\delta ,\mu )=c(\lambda ,\alpha ,\beta ,\delta )\times \frac{K_{\lambda -1/2}(\alpha \sqrt{\delta^{2}+{\left(x-\mu \right)}^{2}})}{{\left(\sqrt{\delta^{2}+{\left(x-\mu \right)}^{2}}/\alpha \right)}^{1/2-\lambda }}e^{\beta (x-\mu )}$$ (11)

The density function of GH distribution can also be formulated as:

GH=f(λ,α,β,δ,μ) (12)

The properties of GH distribution were mainly determined by five parameters *α*, *β*, *μ*, *λ* and *δ*. Among these parameters, *α* and *β* indicated the kurtosis and skewness of the distribution respectively; *μ* and *δ* indicated the position and shape of density function; and *λ* indicated the tail thickness of the distribution. In step two, by calling the ryperb function in *R*, three groups of skewed data were generated for the *p×i* design. The parameters of GH distribution were controlled by setting *α* = 1, *μ* = 0, *δ* = 1, *α* = 3; *β* was free and could be set at –2, –1, 0, 1, and 2. The result of *β* was symmetrical, and *β* was only set at –2, –1, and 0. The skewed data under certain of skewness were generated by using the following equation: $X_{pi}=\mu +GH(p)+GH(i)+GH(pi)$ (usually *μ* set as 0). In step three, for certain skewness, the simulated skewed data were matrix data (*p×i*), and the number of simulations was 1000. Thus, 1000 batches of 100×20 skewed data were simulated. There were three skewness values (–2, –1, and 0), which could generate 3×1000 batches of 100×20 skewed data.

### 2.4 Measurement effect

In this paper, three measurement effects are considered, such as person effect (*p* effect), item (*i* effect), and person×item (*pi*,*e* effect). The effect of person×item includes residual effects that are considered as some random and inseparable effects [33].

### 2.5 Comparison standard

Following the recommendations of Diallo et al. (2017) [34], this study uses the Relative Percentage Bias (RPB) as the standard of comparison in estimating the variance components and their variability. The Bias and RPB are formulated as:

$$RPB=\frac{\hat{\theta }-\theta }{\theta }\times 100$$ (13)

Where the RPB is the relative percentage deviation, $\hat{\theta }$ is the estimated value of the variance component and the variability, and *θ* is the parameter value of the variance component and the variability.

A smaller absolute value of the relative percentile deviation (|RPB|) indicates a smaller difference between the estimated value and the parameter value, and that the result would be more reliable, and vice versa.

Following the recommendations of Tong and Brennan (2007) [13], the following decision rules are determined: (1) If |RPB| < 25, the deviation is relatively small and the estimation is considered as accurate and reliable; (2) If |RPB| ≥ 25, the deviation is relatively large and the result is considered as inaccurate and unreliable.

### 2.6 Analytical tools

Several statistical programs, including *R*, *WinBUGS*, *R2WinBUGS*, and *CODA*, were used in the current study. The analyses were completed without interruptions.

## 3 Results

### 3.1 Estimating variance components and variability

Tables 1 and 2 shows the estimates of variance components and corresponding variability based on the *p×i* design. This variability refers to the standard errors and confidence intervals. The first column displays four types of data, including normal, dichotomous, polytomous and skewed data (low skew, *β* = 0; medium skew, *β* = –1; high skew, *β* = –2). The second column displays four methods used, including the traditional method, jackknife method, bootstrap method, and Markov Chain Monte Carlo method. For the bootstrap method, six resampling strategies were considered, including boot-pi, boot-pir, boot-ir, boot-i, boot-pr, and boot-p. The third column displays estimated variance components for person. The fourth column displays the standard errors of estimated variance components for person. The fifth column displays the confidence intervals of estimated variance components for person and so on. It is worth noting that in Tables 1 and 2. The parameter values are given in the first row corresponding to each distribution.

**Table 1:** Estimated variance components and the variability for normal, dichotomous and polytomous distribution.

Distribution	Method	vc.p	SE (vc.p)	CI (vc.p)	vc.i	SE (vc.i)	CI (vc.i)	vc.pi	SE (vc.pi)	CI (vc.pi)
Normal	Parameter	4.0000	1.0287	0.8000	16.0000	5.3988	0.8000	64.0000	2.0869	0.8000
	traditional	4.0284	1.0323	0.7920	16.0835	5.4257	0.8260	63.9494	2.0852	0.8080
	jackknife	4.0152	0.9981	0.7610	16.0913	5.2304	0.7660	63.9903	2.0390	0.7750
	boot-pi	4.031	2.0963	0.9840	16.083	5.3965	0.8020	63.9510	3.7028	0.9660
	boot-pir	4.0298	1.4584	0.9150	16.0782	5.1803	0.7850	63.9483	2.083	0.8110
	boot-ir	4.0285	1.0484	0.7900	16.0805	5.1807	0.7970	63.9483	2.0833	0.8000
	boot-i	4.0291	1.4974	0.9040	16.0788	4.9500	0.7810	63.9482	2.2951	0.8460
	boot-pr	4.0298	1.4584	0.9200	16.0834	1.4493	0.2770	63.9483	2.0830	0.8070
	boot-p	4.0289	1.0095	0.7740	16.0855	1.4893	0.2930	63.9507	2.0697	0.8040
	MCMC	3.8219	0.9947	0.7690	16.0062	5.4475	0.7950	64.0304	2.0688	0.7900
Dichotomous	Parameter	0.0125	0.0043	0.8000	0.0025	0.0007	0.8000	0.2500	0.0103	0.8000
	traditional	0.0126	0.0036	0.7080	0.0025	0.0016	0.9890	0.2499	0.0081	0.7050
	jackknife	0.0122	0.0042	0.7640	0.0025	0.0004	0.4760	0.2480	0.0058	0.5140
	boot-pi	0.0126	0.0102	0.9940	0.0025	0.0008	0.8230	0.2499	0.0125	0.8920
	boot-pir	0.0126	0.0065	0.9400	0.0025	0.0008	0.8470	0.2499	0.0052	0.5040
	boot-ir	0.0125	0.0051	0.8620	0.0025	0.0008	0.8280	0.2499	0.0052	0.4950
	boot-i	0.0126	0.0078	0.9500	0.0025	0.0007	0.7830	0.2499	0.0104	0.8150
	boot-pr	0.0125	0.0065	0.9340	0.0025	0.0003	0.4110	0.2499	0.0052	0.5030
	boot-p	0.0125	0.0041	0.7730	0.0025	0.0003	0.3890	0.2499	0.0048	0.4700
	MCMC	0.0112	0.0038	0.7380	0.0025	0.0009	0.8490	0.2504	0.0081	0.6800
Polytomous	Parameter	0.5020	0.1076	0.8000	0.2240	0.1889	0.8000	1.2080	0.0356	0.8000
	traditional	0.4987	0.0795	0.6680	0.2189	0.0750	0.3870	1.2070	0.0394	0.8490
	jackknife	0.5026	0.1066	0.7840	0.2183	0.1590	0.6791	1.2100	0.0349	0.7530
	boot-pi	0.4988	0.1191	0.8360	0.2187	0.1512	0.6820	1.2070	0.0645	0.9770
	boot-pir	0.4988	0.1114	0.8120	0.2187	0.1491	0.6880	1.2070	0.0362	0.7970
	boot-ir	0.4987	0.0368	0.3320	0.2188	0.1492	0.6700	1.2070	0.0362	0.8060
	boot-i	0.4988	0.0416	0.3790	0.2187	0.1466	0.6260	1.2071	0.0404	0.8430
	boot-pr	0.4988	0.1114	0.8250	0.2190	0.0213	0.2100	1.2070	0.0362	0.8010

**Table 2:** Estimated variance components and the variability for skewed distribution.

Skewed distribution	Method	vc.p	SE (vc.p)	CI (vc.p)	vc.i	SE (vc.i)	CI (vc.i)	vc.pi	SE (vc.pi)	CI (vc.pi)
*β* = 0	Parameter	0.5080	0.0887	0.8000	0.5171	0.2003	0.8000	0.5106	0.0203	0.8000
	traditional	0.5114	0.0763	0.7310	0.5097	0.1670	0.7590	0.5108	0.0167	0.7000
	jackknife	0.5152	0.0884	0.7920	0.5141	0.1789	0.7330	0.5113	0.0195	0.7730
	boot-pi	0.5113	0.093	0.8110	0.5097	0.1736	0.7060	0.5108	0.0344	0.9700
	boot-pir	0.5113	0.0896	0.7900	0.5097	0.1720	0.7020	0.5108	0.0195	0.7680
	boot-ir	0.5114	0.0231	0.2350	0.5097	0.1720	0.7310	0.5108	0.0195	0.7660
	boot-i	0.5114	0.0244	0.2450	0.5097	0.1703	0.7240	0.5108	0.0207	0.7860
	boot-pr	0.5113	0.0896	0.7830	0.5097	0.0225	0.1240	0.5108	0.0195	0.7610
	boot-p	0.5114	0.0865	0.7660	0.5097	0.0231	0.1220	0.5108	0.0197	0.7690
	MCMC	0.5065	0.0761	0.7110	0.5105	0.1705	0.7160	0.5107	0.0163	0.6770
*β* = –1	Parameter	0.6456	0.1196	0.8000	0.6543	0.2723	0.8000	0.6491	0.0275	0.8000
	traditional	0.6429	0.0960	0.7110	0.6453	0.2115	0.7450	0.6487	0.0212	0.6680
	jackknife	0.6462	0.1156	0.7820	0.6300	0.2248	0.7030	0.6467	0.0260	0.7588
	boot-pi	0.6431	0.1215	0.8060	0.6454	0.2215	0.7260	0.6487	0.0462	0.9650
	boot-pir	0.6431	0.1173	0.7930	0.6454	0.2195	0.7130	0.6488	0.0263	0.7670
	boot-ir	0.6429	0.0292	0.2460	0.6454	0.2195	0.7300	0.6488	0.0263	0.7770
	boot-i	0.6429	0.0308	0.2640	0.6454	0.2173	0.7330	0.6487	0.0277	0.7820
	boot-pr	0.6431	0.1173	0.7910	0.6453	0.0285	0.1260	0.6488	0.0263	0.7730
	boot-p	0.6431	0.1135	0.7780	0.6453	0.0292	0.1310	0.6487	0.0266	0.7700
	MCMC	0.6369	0.0957	0.7260	0.6463	0.2158	0.8100	0.6486	0.0207	0.6460
*β* = –2	Parameter	1.6634	0.3705	0.8000	1.6723	0.8130	0.8000	1.6613	0.0810	0.8000
	traditional	1.6564	0.2472	0.6300	1.6932	0.5547	0.6460	1.6580	0.0541	0.6420
	jackknife	1.6824	0.3456	0.7610	1.6628	0.6542	0.6460	1.6562	0.0792	0.7690
	boot-pi	1.6566	0.3526	0.7670	1.6935	0.6479	0.6720	1.6580	0.1371	0.9530
	boot-pir	1.6566	0.3429	0.7520	1.6934	0.6427	0.6730	1.6580	0.0783	0.7930
	boot-ir	1.6564	0.0746	0.2100	1.6935	0.6427	0.6740	1.6580	0.0783	0.7940
	boot-i	1.6565	0.0792	0.2300	1.6935	0.6375	0.6750	1.6580	0.0810	0.8120
	boot-pr	1.6566	0.3429	0.7640	1.6931	0.0731	0.0870	1.6580	0.0783	0.7970
	boot-p	1.6566	0.3339	0.7400	1.6931	0.0749	0.0870	1.6580	0.0800	0.8100
	MCMC	1.6411	0.2466	0.6140	1.6944	0.5655	0.6070	1.6578	0.0530	0.6100

When only the sampling of *p* (person) is considered, boot-p represents fixed *i* (item) and r(residual); when only the sampling of *i* is considered, boot-i represents fixed *p* and *r*; when only the sampling of *p* and *i* are considered, boot-pi represents fixed *r*; when only the sampling of *p* and *r* are considered, boot-pr represents fixed *i*; when the sampling of *i* and *r* are considered, boot-ir represents fixed *p*. When the sampling of *p*, *i* and *r* are considered, boot-pir represents the sampling of *p*, *i*, and *r*, simultaneously. Parameter is the parameter value.

The abbreviations of “vc.p”, “vc.i”, and “vc.pi” are short for variance components of the person, the item, and the interaction between the person and the item (including the residual) respectively. SE(vc.p), SE(vc.i), and SE(vc.pi) are their corresponding standard errors. CI (vc.p), CI (vc.i), and CI (vc.pi) represent the 80% confidence intervals of the estimates (80% CI).

The parameter values from Tables 1 and 2 were converted using the RPB conversion formula (i.e., Formula (13)) and displayed in Tables 3 and 4.

**Table 3:** The relative percent deviation after conversion of estimated variance components and the variability for normal, dichotomous and polytomous distribution.

Distribution	Method	vc.p	SE (vc.p)	CI (vc.p)	vc.i	SE (vc.i)	CI (vc.i)	vc.pi	SE (vc.pi)	CI (vc.pi)
Normal	traditional	0.7100	0.3499	-1.0000	0.5219	0.4983	3.2500	-0.0791	-0.0815	1.0000
	jackknife	0.3800	-2.9746	-4.8750	0.5706	-3.1192	-4.2500	-0.0152	-2.2953	-3.1250
	boot-pi	0.7750	103.7815	*23*.*0000*	0.5187	*-0*.*0426*	*0*.*2500*	-0.0766	77.4306	*20*.*7500*
	boot-pir	0.7450	41.7712	*14*.*3750*	0.4887	*-4*.*0472*	*-1*.*8750*	-0.0808	*-0*.*1869*	*1*.*3750*
	boot-ir	0.7125	*1*.*9150*	*-1*.*2500*	0.5031	*-4*.*0398*	*-0*.*3750*	-0.0808	*-0*.*1725*	*0*.*0000*
	boot-i	0.7275	45.5624	*13*.*0000*	0.4925	*-8*.*3130*	*-2*.*3750*	-0.0809	*9*.*9765*	*5*.*7500*
	boot-pr	0.7450	41.7712	*15*.*0000*	0.5213	-73.1551	-65.3750	-0.0808	*-0*.*1869*	*0*.*8750*
	boot-p	0.7225	-1.8664	*-3*.*2500*	0.5344	-72.4142	-63.3750	-0.0770	*-0*.*8242*	*0*.*5000*
	MCMC	-4.4525	-3.3051	-3.8750	0.0387	0.9021	-0.6250	0.0475	-0.8673	-1.2500
Dichotomous	traditional	0.8000	-16.2791	-11.5000	0.0000	**128.5714**	23.6250	-0.0400	-21.3592	-11.8750
	jackknife	-2.4000	-2.3256	-4.5000	0.0000	**-42.8571**	**-40.5000**	-0.8000	**-43.6893**	**-35.7500**
	boot-pi	0.8000	137.2093	***24*.*2500***	0.0000	***14*.*2857***	***2*.*8750***	-0.0400	***21*.*3592***	***11*.*5000***
	boot-pir	0.8000	51.1628	***17*.*5000***	0.0000	***14*.*2857***	***5*.*8750***	-0.0400	-49.5146	-37.0000
	boot-ir	0.0000	*18*.*6047*	***7*.*7500***	0.0000	***14*.*2857***	***3*.*5000***	-0.0400	-49.5146	-38.1250
	boot-i	0.8000	81.3953	***18*.*7500***	0.0000	***0*.*0000***	***-2*.*1250***	-0.0400	***0*.*9709***	***1*.*8750***
	boot-pr	0.0000	51.1628	***16*.*7500***	0.0000	-57.1429	-48.6250	-0.0400	-49.5146	-37.1250
	boot-p	0.0000	***-4*.*6512***	***-3*.*3750***	0.0000	-57.1429	-51.3750	-0.0400	-53.3981	-41.2500
	MCMC	-10.4000	-11.6279	-7.7500	0.0000	**28.5714**	6.1250	0.1600	-21.3592	-15.0000
Polytomous	traditional	-0.6574	**-26.1152**	-16.5000	-2.2768	**-60.2965**	**-51.6250**	-0.0828	10.6742	6.1250
	jackknife	0.1195	-0.9294	-2.0000	-2.5446	-15.8285	-15.1125	0.1656	-1.9663	-5.8750
	boot-pi	-0.6375	***10*.*6877***	***4*.*5000***	-2.3661	***-19*.*9576***	***-14*.*7500***	-0.0828	81.1798	***22*.*1250***
	boot-pir	-0.6375	***3*.*5316***	***1*.*5000***	-2.3661	***-21*.*0693***	***-14*.*0000***	-0.0828	***1*.*6854***	***-0*.*3750***
	boot-ir	-0.6574	-65.7993	-58.5000	-2.3214	***-21*.*0164***	***-16*.*2500***	-0.0828	***1*.*6854***	***0*.*7500***
	boot-i	-0.6375	-61.3383	-52.6250	-2.3661	***-22*.*3928***	***-21*.*7500***	-0.0745	***13*.*4831***	***5*.*3750***
	boot-pr	-0.6375	***3*.*5316***	***3*.*1250***	-2.2321	-88.7242	-73.7500	-0.0828	***1*.*6854***	***0*.*1250***
	boot-p	-0.6375	***-2*.*3234***	***-0*.*7500***	-2.2321	-88.4066	-73.0000	-0.0828	***0*.*0000***	***-1*.*2500***
	MCMC	-1.5538	**-26.0223**	-14.8750	-1.3393	**-59.6612**	**-45.2500**	-0.1325	8.4270	1.8750

**Table 4:** The relative percent deviation after conversion of estimated variance components and the variability for skewed distribution.

Skewed distribution	Method	vc.p	SE (vc.p)	CI (vc.p)	vc.i	SE (vc.i)	CI (vc.i)	vc.pi	SE (vc.pi)	CI (vc.pi)
*β* = 0	traditional	0.6693	-13.9797	-8.6250	-1.4311	-16.6251	-5.1250	0.0392	-17.7340	-12.5000
	jackknife	1.4173	-0.3382	-1.0000	-0.5802	-10.6840	-8.3750	0.1336	-3.9409	-3.3750
	boot-pi	0.6496	***4*.*8478***	***1*.*3750***	-1.4311	***-13*.*3300***	***-11*.*7500***	0.0392	69.4581	***21*.*2500***
	boot-pir	0.6496	***1*.*0147***	***-1*.*2500***	-1.4311	***-14*.*1288***	***-12*.*2500***	0.0392	***-3*.*9409***	***-4*.*0000***
	boot-ir	0.6693	-73.9572	-70.6250	-1.4311	***-14*.*1288***	***-8*.*6250***	0.0392	***-3*.*9409***	***-4*.*2500***
	boot-i	0.6693	-72.4915	-69.3750	-1.4311	***-14*.*9775***	***-9*.*5000***	0.0392	***1*.*9704***	***-1*.*7500***
	boot-pr	0.6496	***1*.*0147***	***-2*.*1250***	-1.4311	-88.7668	-84.5000	0.0392	***-3*.*9409***	***-4*.*8750***
	boot-p	0.6693	***-2*.*4803***	***-4*.*2500***	-1.4311	-88.4673	-84.7500	0.0392	***-2*.*9557***	***-3*.*8750***
	MCMC	-0.2953	-14.2052	-11.1250	-1.2763	-14.8777	-10.5000	0.0196	-19.7044	-15.3750
*β* = –1	traditional	-0.4182	-19.7324	-11.1250	-1.3755	-22.3283	-6.8750	-0.0616	-22.9091	-16.5000
	jackknife	0.0929	-3.3445	-2.2500	-3.7139	-17.4440	-12.1250	-0.3697	-5.4545	-5.1500
	boot-pi	-0.3872	***1*.*5886***	***0*.*7500***	-1.3602	***-18*.*6559***	***-9*.*2500***	-0.0616	68.0000	***20*.*6250***
	boot-pir	-0.3872	***-1*.*9231***	***-0*.*8750***	-1.3602	***-19*.*3904***	***-10*.*8750***	-0.0462	***-4*.*3636***	***-4*.*1250***
	boot-ir	-0.4182	-75.5853	-69.2500	-1.3602	***-19*.*3904***	***-8*.*7500***	-0.0462	***-4*.*3636***	***-2*.*8750***
	boot-i	-0.4182	-74.2475	-67.0000	-1.3602	***-20*.*1983***	***-8*.*3750***	-0.0616	***0*.*7273***	***-2*.*2500***
	boot-pr	-0.3872	***-1*.*9231***	***-1*.*1250***	-1.3755	-89.5336	-84.2500	-0.0462	***-4*.*3636***	***-3*.*3750***
	boot-p	-0.3872	***-5*.*1003***	***-2*.*7500***	-1.3755	-89.2765	-83.6250	-0.0616	***-3*.*2727***	***-3*.*7500***
	MCMC	-1.3476	-19.9833	-9.2500	-1.2227	-20.7492	1.2500	-0.0770	-24.7273	-19.2500
*β* = –2	traditional	-0.4208	**-33.2794**	-21.2500	1.2498	**-31.7712**	-19.2500	-0.1986	**-33.2099**	-19.7500
	jackknife	1.1422	-6.7206	-4.8750	-0.5681	-19.5326	-19.2500	-0.3070	-2.2222	-3.8750
	boot-pi	-0.4088	***-4*.*8313***	***-4*.*1250***	1.2677	***-20*.*3075***	***-16*.*0000***	-0.1986	69.2593	***19*.*1250***
	boot-pir	-0.4088	***-7*.*4494***	***-6*.*0000***	1.2617	***-20*.*9471***	***-15*.*8750***	-0.1986	***-3*.*3333***	***-0*.*8750***
	boot-ir	-0.4208	-79.8650	-73.7500	1.2677	***-20*.*9471***	***-15*.*7500***	-0.1986	***-3*.*3333***	***-0*.*7500***
	boot-i	-0.4148	-78.6235	-71.2500	1.2677	***-21*.*5867***	***-15*.*6250***	-0.1986	***0*.*0000***	***1*.*5000***
	boot-pr	-0.4088	***-7*.*4494***	***-4*.*5000***	1.2438	-91.0086	-89.1250	-0.1986	***-3*.*3333***	***-0*.*3750***
	boot-p	-0.4088	***-9*.*8785***	***-7*.*5000***	1.2438	-90.7872	-89.1250	-0.1986	***-1*.*2346***	***1*.*2500***
	MCMC	-1.3406	**-33.4413**	-23.2500	1.3215	**-30.4428**	-24.1250	-0.2107	**-34.5679**	-23.7500

For example, for Table 3, How to obtain 0.7100 for traditional method in normal data? First of all, we should see Table 1 and find 4.0284 in column vc.p, and also see the corresponding parameter 4.0000 in Table 1. Secondly, we use Formula (13) to compute as follows:

$$RPB=\frac{\hat{\theta }-\theta }{\theta }\times 100=\frac{4.0284-4.0000}{4.0000}=0.007100\times 100=0.7100$$

The 0.7100 is in Table 3, which is the Relative Percentage Bias of vc.p of traditional method in normal data.

Similarly, we can compute the Relative Percentage Bias of SE(vc.p) of traditional method in normal data.


$$RPB=\frac{\hat{\theta }-\theta }{\theta }\times 100=\frac{1.0323-1.0287}{1.0287}=0.003499\times 100=0.3499$$

Similarly, again, we can also compute the Relative Percentage Bias of vc.p and SE(vc.p) of traditional method in skewed data (*β* = 0).

$$RPB=\frac{\hat{\theta }-\theta }{\theta }\times 100=\frac{0.5114-0.5080}{0.5080}=0.006693\times 100=0.6693$$

$$RPB=\frac{\hat{\theta }-\theta }{\theta }\times 100=\frac{0.0763-0.0887}{0.0887}=-0.139797\times 100=-13.9797$$

Accordingly, as shown in the boxed areas of Tables 3 and 4, the traditional, jackknife, and MCMC procedures fail to provide accurate estimates (|RPB| ≥ 25). When dealing with the dichotomous data, the traditional method cannot estimate the standard errors of the variance components accurately, as |RPB| of SE (vc.i) is 128.5714, which is larger than 25. When dealing with the dichotomous data, the jackknife method cannot estimate the standard errors of the variance components accurately, as the |RPB| of SE (vc.i) is 42.8571, the |RPB| of CI (vc.i) is 40.5000 and the |RPB| of SE (vc.pi) is 43.6893, which are larger than 25. When dealing with the polytomous data, the MCMC method cannot estimate the standard errors [SE (vc.i), |RPB| = 59.6612 ≥ 25] or the confidence intervals [CI (vc.i), |RPB| = 45.2500 ≥ 25] of the variance components accurately. However, the bootstrap procedure succeeds in providing accurate results, regardless of which one of the six resampling strategies is adopted (see the italic bold values; |RPB| < 25).

### 3.2 Comparing the performance of estimation methods with different types of data

Based on the standard of comparison and decision rules, the performance of these methods under different data conditions is graded and showed in Table 5, with the "+" symbol meaning accurate and the "–" symbol meaning inaccurate. As shown in Table 5, the traditional method, jackknife method and MCMC method are inaccurate under certain conditions. To be specific, when using the traditional method, SE (vc.i) obtained from dichotomous data, SE (vc.p), SE (vc.i) and CI (vc.i) obtained from polytomous data, and SE (vc.p), SE (vc.i) and SE(vc.pi) obtained from skewed data (high skewness) are estimated inaccurately (|RPB| ≥ 25). Regarding the jackknife method, SE (vc.i), SE (vc.pi), CI (vc.i), and CI (vc.pi) are inaccurate for dichotomous data. As for the MCMC method, SE (vc.i) obtained from dichotomous data, SE (vc.p), SE (vc.i) and CI (vc.i) obtained from polytomous data, and SE (vc.p), SE (vc.i) and SE (vc.pi) obtained from highly skewed data are inaccurate (with |RPB| ≥ 25). Compared with the above-mentioned methods, the bootstrap method has much better performance. No matter which one of the six resampling strategies is chosen, the estimated variance components are accurate, and the standard errors and the 80% CI of estimated variance components contain a total of 2 to 6 resampling strategies. Furthermore, regardless of data types, the bootstrap method can always produce accurate estimated variance components and their variability with a certain resampling strategy.

**Table 5:** Comparison of the performance of the four methods under the four distributions data.

Distribution	Variance components and their variability	Traditional^1^	Jackknife^2^	Bootstrap^3^	MCMC^4^
Normal	Variance Components	+	+	+	+
	Standard Errors	+	+	+	+
	Confidence Intervals	+	+	+	+
Dichotomous	Variance Components	+	+	+	+
	Standard Errors	–	–	+	–
	Confidence Intervals	+	–	+	+
Polytomous	Variance Components	+	+	+	+
	Standard Errors	–	+	+	–
	Confidence Intervals	–	+	+	–
*β* = 0	Variance Components	+	+	+	+
	Standard Errors	+	+	+	+
	Confidence Intervals	+	+	+	+
*β* = –1	Variance Components	+	+	+	+
	Standard Errors	+	+	+	+
	Confidence Intervals	+	+	+	+
*β* = –2	Variance Components	+	+	+	+
	Standard Errors	–	+	+	–
	Confidence Intervals	+	+	+	+
	**Rank**	**fourth**	**second**	**first**	**third**

**Note:** ^1^only refers to the revised Traditional method [4]; ^2^only refers to the synthesized Jackknife method [14]; ^3^only refers to the corrected Bootstrap method [12]; ^4^only refers to the MCMC method [29].

In sum, the bootstrap method has shown the best estimation performance when applied to all four types of data in estimating variance components and the variability (no "–" was produced). The jackknife method is better (with two "–") than the traditional and the MCMC methods (both with four "–"). The results show that the traditional method produced a larger total error (|RPB| = 734.3263) than the MCMC method (|RPB| = 633.1782). Therefore, compared with the traditional method, the MCMC method is a better option to estimate the variability of estimated variance components.

### 3.3 Divide-and-conquer strategy of the bootstrap method with different data

Although the bootstrap method is optimal for estimating the variability of estimated variance components, it requires a divide-and-conquer strategy [13]. That is, the resampling strategies of the bootstrap method should be chosen appropriately based on specific variability of the variance components. We have tested the divide-and-conquer strategy (based on Table 4) for different standard errors and confidence intervals of the variance components with different distributions of data, and results are summarized in Table 6. For normal data, SE (vc.p) can be estimated with boot-p and boot-ir (|RPB| < 25; 1.8664 and 1.9150, respectively; Table 4); boot-pi, boot-ir, boot-pir, and boot-i can be used to estimate SE (vc.i) (all |RPB| < 25); SE (vc.pi) can be estimated with boot-ir, boot-pir, boot-pr, boot-p and boot-i (all |RPB| < 25). In the case of normally distributed data, all six bootstrap strategies can be used to estimate CI (vc.p). With respect to the 80% coverage of confidence intervals, |RPB| of boot-ir, boot-p, boot-i, boot-pir, boot-pr, and boot-pi are all less than 25 (1.2500, 3.2500, 13.0000, 14.3750, 15.0000, and 23.0000 respectively). CI (vc.i) can be estimated with boot-pi, boot-ir, boot-pir, and boot-i (all |RPB| < 25). All six strategies can be chosen for the estimation of CI (vc.pi). See Table 6 and Table 6 to review the bootstrap strategies for the estimation of standard errors and confidence intervals of the estimates under other distribution data.

**Table 6:** The divide-and-conquer strategy for estimating the variablility of different data variance components with the bootstrap method.

Variability	Distribution	vc.p	vc.i	vc.pi
Standard Errors	Normal	**boot-p**, boot-ir	**boot-pi**, boot-ir, boot-pir, boot-i	boot-ir, boot-pir, boot-pr, boot-p, **boot-i**
	Dichotomous	**boot-p**, boot-ir	boot-i, **boot-pi**, boot-pir, boot-ir	**boot-i**, boot-pi
	Polytomous	**boot-p**, boot-pr, boot-pir, boot-pi	**boot-pi**, boot-ir, boot-pir, boot-i	boot-p, boot-pr, boot-pir, boot-ir, **boot-i**
	*β* = 0	boot-pr, boot-pir, **boot-p**, boot-pi	**boot-pi**, boot-ir, boot-pir, boot-i	**boot-i**, boot-p, boot-pr, boot-ir, boot-pir
	*β* = –1	boot-pi, boot-pr, boot-pir, **boot-p**	**boot-pi**, boot-ir, boot-pir, boot-i	**boot-i**, boot-p, boot-pr, boot-ir, boot-pir
	*β* = –2	boot-pi, boot-pr, boot-pir, **boot-p**	**boot-pi**, boot-ir, boot-pir, boot-i	**boot-i**, boot-p, boot-pr, boot-ir, boot-pir
	**Optimal Strategy**	**boot-p**	**boot-pi**	**boot-i**
Confidence Intervals	Normal	boot-ir, **boot-p**, boot-i, boot-pir, boot-pr, boot-pi	**boot-pi**, boot-ir, boot-pir, boot-i	boot-ir, boot-p, boot-pr, boot-pir, **boot-i**, boot-pi
	Dichotomous	**boot-p**, boot-ir, boot-pr, boot-pir, boot-I, boot-pi	boot-i, **boot-pi**, boot-ir, boot-pir	**boot-i**, boot-pi
	Polytomous	**boot-p**, boot-pir, boot-pr, boot-pi	boot-pir, **boot-pi**, boot-ir, boot-i	boot-pr, boot-pir, boot-ir, boot-p, **boot-i**, boot-pi
	*β* = 0	boot-pir, boot-pi, boot-pr, **boot-p**	boot-ir, boot-i, **boot-pi**, boot-pir	**boot-i**, boot-p, boot-pir, boot-ir, boot-pr, boot-pi
	*β* = –1	boot-pi, boot-pir, boot-pr, **boot-p**	boot-i, boot-ir, **boot-pi**, boot-pir	**boot-i**, boot-ir, boot-pr, boot-p, boot-pir, boot-pi
	*β* = –2	boot-pi, boot-pr, boot-pir, **boot-p**	boot-i, boot-ir, boot-pir, **boot-pi**	boot-pr, boot-ir, boot-pir, boot-p, **boot-i**, boot-pi
	**Optimal Strategy**	**boot-p**	**boot-pi**	**boot-i**

## 4 Discussions

### 4.1 The cross-distribution superiority of the bootstrap method

As showed in Table 5, for the normal data, all four methods can accurately estimate the variance components, the standard errors of variance components, and the confidence intervals of variance components ("+"). Specifically, when using the bootstrap method, the divide-and-conquer strategy should be adopted.

For dichotomous data, all four methods perform well in terms of the estimation of the variance components. However, when estimating the standard errors of variance components, only one method, the bootstrap method using the divide-and-conquer strategy can produce accurate outcomes. Both the traditional method and the MCMC method overestimate the standard errors of the variance components of *i* (by 128.5714% and 28.5714% respectively), and the jackknife method underestimates the standard errors of the variance components of *i* and *pi* (by 42.8571% and 43.6893% respectively). Regarding the estimation of the confidence intervals of variance components, both the MCMC method and the bootstrap method (using the divide-and-conquer strategy) produce accurate outcomes. The traditional method performs relatively accurately as well. Nonetheless, the jackknife method underestimates the 80% coverage of the confidence intervals of variance components of *i* and *pi* (by 40.5000% and 35.7500%).

For polytomous data, all four methods can estimate the variance components accurately. When estimating the standard errors of the variance components, the jackknife method and the bootstrap method (when adopting the divide-and-conquer strategy) can estimate the standard errors accurately, while the traditional method and the MCMC method underestimate the standard errors of the variance components of *p* (by 26.1152% and 26.0223% respectively) and *i* (by 60.2965% and 59.6612% respectively). In the estimation of the confidence intervals of variance components, both the jackknife method and the bootstrap method (with the divide-and-conquer strategy) have good performance whereas the traditional method and the MCMC method underestimate the 80% coverage of the confidence intervals of variance components of *i* (by 51.6250% and 45.2500%, respectively).

For skewed data, in the cases of low skewness (*β* = 0) and medium skewness (*β* = –1), all four methods can accurately estimate the variance components, the standard errors of variance components, and the confidence intervals of variance components. Similar to previous results, the bootstrap method performs well when the divide-and-conquer strategy is used. In the case of high skewness (*β* = –2), all methods are accurate in estimating the variance components. In terms of estimating the standard errors of variance components, the jackknife method and the bootstrap method (when using the divide-and-conquer strategy) perform accurately, but the traditional method and the MCMC method underestimate the standard errors of the variance components of *p* (by 33.2794% and 33.4413%), *i* (31.7712% and 30.4428%) and *pi* (33.2099% and 34.5679%). As for the estimation of the confidence intervals of variance components, all methods yield accurate outcomes. Again, the bootstrap method can be accurate when the divide-and-conquer strategy is used.

In sum, firstly, all four methods can accurately estimate these three variance components (i.e., vc.p, vc.i, vc.pi). This lays the basis for estimating and comparing the performance of the corresponding variability of estimated variance components using various methods. Secondly, all four estimation methods have an impact on the variability of estimated variance components, and their performance depends on which type of data is applied to. Specifically, for normal data, all four methods are acceptable. For dichotomous data, the bootstrap method is the best option, as the other three methods perform poorly. For polytomous data, the bootstrap method and jackknife method have better performance than the traditional method and MCMC method. Likewise, for skewed data, the bootstrap method and jackknife methods are superior to the traditional method and MCMC method. Thirdly, there are differences in the overall performance of these estimation methods in estimating the variability of the variance components. The bootstrap method is the best option, followed by the jackknife method, the MCMC method, and the traditional method. Finally, only the bootstrap method can accurately estimate the variability of the variance components for all four types of data, showing superiority over the other methods across different data conditions. It should be noted that when using the bootstrap method, the divide-and conquer strategy should be chosen.

### 4.2 The unified rule of the bootstrap method

Since the bootstrap method is the only method that performs well with different types of data in the study, we argue that it provides convenience for estimating the variability of estimated variance components. When using a divide-and-conquer strategy, the bootstrap method produces accurate results regardless of the data type. However, one problem remains to be solved. Does the divide-and-conquer strategy vary according to the distributions of data? In other words, does a set of unified rules exist?

#### 4.2.1 The unified rule for estimating the standard errors of variance components using the bootstrap method

In terms of the standard errors of vc.p (see Table 6), only boot-p and boot-ir strategies can be selected for normal data and dichotomous data. However, boot-ir does not appear in other types of data. Only boot-p has “spanning” in all four types of data and shows good performance. Thus, the boot-p strategy is the best estimation strategy for the standard errors of vc.p. For the standard errors of vc.i, the boot-pi strategy performs best, except for its performance with dichotomous data (ranked second). It is obvious that the boot-pi strategy is the best estimation strategy for the standard errors of vc.i. For the standard errors of vc.pi, only the boot-i and boot-pi strategies can be selected for dichotomous data. However, boot-pi only appears in the dichotomous data. Only boot-i can be used uniquely. The boot-i has "spanning" in all four types of data. As a result, the boot-i strategy is the best estimation strategy for the standard errors of vc.pi. To sum up, the boot-p strategy is optimal for estimating the standard errors of vc.p, the boot-pi strategy is optimal for *i*, and the boot-i strategy is optimal for *pi*. It should be noted that the unified rules for estimating the standard errors of variance components of the bootstrap method in this study are of great significance and can provide guidance for future use of the bootstrap method [13].

#### 4.2.2 The unified rule for estimating the confidence intervals of the variance components using the bootstrap method

In terms of the confidence intervals of vc.p (see Table 6), four strategies of boot-p, boot-pi, boot-pr, and boot-pir are applied to four types of data. According to the absolute "relative percent deviations" (|RPB| = 21.8750, 58.0000, 42.6250, and 41.5000, respectively, for the boot-p, boot-pi, boot-pr, and boot-pir strategies), the boot-p strategy has the smallest deviation, and it is the best estimation strategy for the confidence intervals of vc.p. For the confidence intervals of vc.i, four strategies (i.e., boot-pi, boot-i, boot-pir, and boot-ir) also appear under all types of data. The absolute "relative percent deviations" of boot-pi, boot-i, boot-pir, and boot-ir strategies are 54.8750, 59.7500, 60.7500, and 53.2500, respectively. The deviation of the boot-pi strategy is relatively small (i.e., 54.8750), and there is no significant difference from the boot-ir strategy, which has a minimum |RPB| (i.e., 53.2500). It can be considered that the boot-pi strategy is the best estimation strategy for the confidence intervals of vc.i. For the confidence intervals of vc.pi, only the boot-i and boot-pi strategies can be selected for the dichotomous distribution data. The absolute "relative percent deviations" of boot-i and boot-pi strategies are 10.5000 and 115.3750, respectively. The deviation of the boot-i strategy is smaller, and the boot-i strategy is the best estimation strategy for the confidence intervals of vc.pi. In summary, the boot-p strategy is optimal for the estimation of the confidence intervals of the variance components of *p*, the boot-pi strategy is the best for *i*, and the boot-i strategy performs best for *pi*. That is, for p, boot-p is the best; for i, boot-pi is the best; and for pi, boot-i is the best.

### 4.3 Limitations

The present study has several limitations. First of all, regarding the data simulation process, the skewed data were simulated without taking various kurtoses, dispersion, or tail thickness into account [31]. In addition, the sample size was fixed at 100×20. Other sample sizes, such as 30×5, 30×20, 600×5, 600×20, 100×40 and 100×80, can be used in future study. Secondly, only the *p×i* design was investigated in this study. Other designs such as *i*:*p*, *i*:*h*:*p*, *p×i×h*, *p×(i*:*h)*, and *i*:*(p×h)* should be examined in future research. Thirdly, this study used the Bias and RPB as the standard of comparison in estimating the variability of estimated variance components. Future study can also include the root mean square error (RMSE) [35] as a standard of comparison. Last but not least, the estimation of D study generalizability coefficients, such as indices of dependability and signal-noise ratios (S-N) for absolute decisions, should be assessed in future study as well.

## 5 Conclusions

In this study, we examined the performance of four methods (i.e., traditional, jackknife, bootstrap, and MCMC) in estimating the variability of estimated variance components with four types of data (i.e., normal, dichotomous, polytomous, and skewed data) and found that these methods have different performance under different conditions. The bootstrap method is the only one that can accurately estimate the variability of variance components with all four types of data, showing cross-distribution superiority over the other methods. When using the bootstrap method, the divide-and-conquer strategy should be used and there is a set of unified rules for this strategy. Specifically, the boot-p strategy is optimal for estimating the variance components and the variability of *p* (person), the boot-pi strategy is optimal for estimating the variance components and the variability of *i* (item), and the boot-i strategy is optimal for estimating the variance components and the variability of *pi* (person × item). That is, boot-p for vc.p, SE(vc.p) and CI(vc.p); boot-pi for vc.i, SE(vc.i) and CI(vc.i); and boot-i for vi.pi, SE(vc.pi) and CI(vc.pi).

## Supporting information

S1 File (RAR)

S1 Data (RAR)
