# Correction: Which method is optimal for estimating variance components and their variability in generalizability theory? Evidence from a set of unified rules for bootstrap method

**DOI:** 10.1371/journal.pone.0336869

**Published:** 2025-11-13

**Authors:** Guangming Li

The word “from” is misspelled as “form” in the article title. The correct title is: Which method is optimal for estimating variance components and their variability in generalizability theory? Evidence from a set of unified rules for bootstrap method. The correct citation is: Li G (2023) Which method is optimal for estimating variance components and their variability in generalizability theory? Evidence from a set of unified rules for bootstrap method. PLoS ONE 18(7): e0288069. https://doi.org/10.1371/journal.pone.0288069
